# A New Weighted Deep Learning Feature Using Particle Swarm and Ant Lion Optimization for Cervical Cancer Diagnosis on Pap Smear Images

**DOI:** 10.3390/diagnostics13172762

**Published:** 2023-08-25

**Authors:** Mohammed Alsalatie, Hiam Alquran, Wan Azani Mustafa, Ala’a Zyout, Ali Mohammad Alqudah, Reham Kaifi, Suhair Qudsieh

**Affiliations:** 1 King Hussein Medical Center, Royal Jordanian Medical Service, The Institute of Biomedical Technology, Amman 11855, Jordan; mhmdsliti312@gmail.com; 2 Department of Biomedical Systems and Informatics Engineering, Yarmouk University, Irbid 21163, Jordan; alzuet@yu.edu.jo (A.Z.); ali_qudah@hotmail.com (A.M.A.); 3 Faculty of Electrical Engineering & Technology, Campus Pauh Putra, Universiti Malaysia Perlis, Arau 02600, Malaysia; 4 Advanced Computing (AdvCOMP), Centre of Excellence (CoE), Universiti Malaysia Perlis, Arau 02600, Malaysia; 5 College of Applied Medical Sciences, King Saud Bin Abdulaziz University for Health Sciences, Jeddah 21423, Saudi Arabia; 6 King Abdullah International Medical Research Center, Jeddah 22384, Saudi Arabia; 7 Department of Obstetrics and Gynecology, Faculty of Medicine, Yarmouk University, Irbid 21163, Jordan; suhair.qudsieh@yu.edu.jo

**Keywords:** Pap smear images, AlexNet, DarkNet-19, NasNet, support vector machine, random forest, cervical cancer, ant lion optimization, particle swarm optimization

## Abstract

One of the most widespread health issues affecting women is cervical cancer. Early detection of cervical cancer through improved screening strategies will reduce cervical cancer-related morbidity and mortality rates worldwide. Using a Pap smear image is a novel method for detecting cervical cancer. Previous studies have focused on whole Pap smear images or extracted nuclei to detect cervical cancer. In this paper, we compared three scenarios of the entire cell, cytoplasm region, or nucleus region only into seven classes of cervical cancer. After applying image augmentation to solve imbalanced data problems, automated features are extracted using three pre-trained convolutional neural networks: AlexNet, DarkNet 19, and NasNet. There are twenty-one features as a result of these scenario combinations. The most important features are split into ten features by the principal component analysis, which reduces the dimensionality. This study employs feature weighting to create an efficient computer-aided cervical cancer diagnosis system. The optimization procedure uses the new evolutionary algorithms known as Ant lion optimization (ALO) and particle swarm optimization (PSO). Finally, two types of machine learning algorithms, support vector machine classifier, and random forest classifier, have been used in this paper to perform classification jobs. With a 99.5% accuracy rate for seven classes using the PSO algorithm, the SVM classifier outperformed the RF, which had a 98.9% accuracy rate in the same region. Our outcome is superior to other studies that used seven classes because of this focus on the tissues rather than just the nucleus. This method will aid physicians in diagnosing precancerous and early-stage cervical cancer by depending on the tissues, rather than on the nucleus. The result can be enhanced using a significant amount of data.

## 1. Introduction

Cervical cancer is a type of cancer that occurs in the cells of the cervix, the lower part of the uterus that connects to the vagina [1]. Worldwide, cervical cancer is the fourth most common cancer in women and the fourth leading cause of cancer-related deaths in women [1,2]. Most cervical cancers take years to progress to a severe stage; early symptoms are often limited to lower back and abdominal pain. Thus, the cancer may go undetected until it is so advanced that it is unresponsive to treatment [3]. The development of cervical carcinoma is preceded by a premalignant epithelial dysplasia called “cervical intraepithelial neoplasia” (CIN). Therefore, the term dysplasia is used to describe the pre-cancerous changes, which are graded using a scale of one to three (mild, moderate, and severe dysplasia) based on how much of the cervical tissue looks abnormal. Precancerous changes can be detected using the Pap test and treated to prevent cancer from developing. Routine screening allows for the identification of precancerous lesions and early-stage cancers when interventions can be most effective. A Pap smear, also known as a Pap test or cervical cytology, is widely used as a screening procedure to detect cellular abnormalities in the cervix, including precancerous or cancerous changes at an early stage.

**Figure 1:**
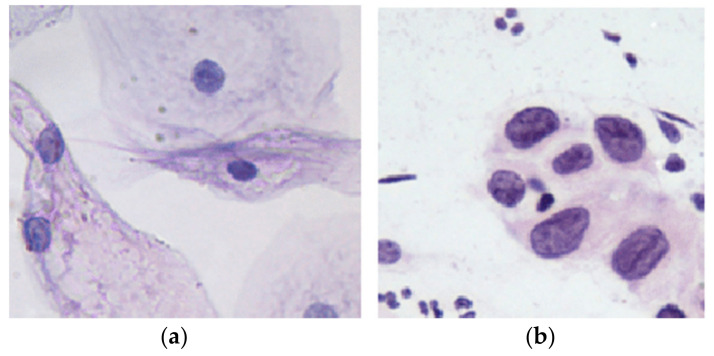
(**a**) Normal Pap smear. (**b**) Abnormal Pap smear 0.201 µm/pixel.

Currently, Pap smears are the most common method for identifying abnormal cervical cells [3,4]. Pap smears are prepared with an Ayre spatula or brush that is used to scrape cells from the cervix. Then, the collected cells are spread over a labeled glass slide before being submerged in 95% ethyl alcohol and delivered to pathology for histopathological analysis to look for abnormal cells that may develop into cancer or observe any inflammation or vaginal infections [5,6]. A cervical cell consists of two main components. One is the nucleus, which is in the cell’s center and surrounded by cytoplasm. The nucleus is typically compact, nearly spherical, darker than the cytoplasm surrounding it, and intense. Dysplastic cells, also known as aberrant cells, are cells that do not develop and divide properly. Dysplastic cells exist at three levels: mild, moderate, and severe. A large percentage of mild dysplastic cells compared to severe dysplastic cells will resolve spontaneously without developing into malignant ones. The nuclei of squamous dysplastic cells are often bigger and darker and frequently stick together in clusters. The nuclei of severe dysplastic cells are often enlarged, covered in black granules, and malformed [7]. Figure 1 shows a sample of normal and abnormal cells.

Pap smear image classification is an important method for diagnosing cervical cancer [8]. By analyzing cell images and categorizing the cells into one of seven classes, Pap smears for advanced cervical screening are a highly effective technique for precancerous cell detection. Computer-aided medical imaging systems have considerably benefited from remarkable advances in artificial intelligence (AI) technology [9]. Thus, this paper aims to take advantage of convolutional neural network (CNN) architectures and machine learning to analyze the classification of cervical Pap smear images to improve the reliability of the test results. Current classification systems use extracted nuclei or full Pap smear images to identify cervical cancer; the main limitation of this approach is that first the nuclei must be detected and excluded from images.

## 2. Literature Review

Many studies have applied computational methods to medical testing to reduce errors in evaluation. Some researchers have investigated the use of traditional machine-learning methods in classifying cervical cells [10]. The computer-assisted detection and classification (CAD) approach for cervical cancer utilizing Pap smear images was proposed by Sukumar et al. [11]. The nuclei cell area was segmented using morphological techniques. From the normal and abnormal cell nucleus, the Grey level, wavelet, and GLCM features were extracted. Using an adaptive neural fuzzy-based classifier (ANFIS) to train and classify these extracted features, they achieved an accuracy of 92.68%. Another study [12] presents the CAD technique for diagnosing cervical cancer utilizing Pap smear images. From Pap smear images, they extract LBP and gray features. The test pap smear cell image was classified into normal or abnormal cell images with a 99.1% accuracy using a hybrid classifier that included SVM and ANFIS. Using morphological techniques, the abnormal cell area was found and segmented.

A new technique for detecting cervical cancer in Pap smear images that show an overlap between the nucleus and inflamed cells was put forth by Muhimmah et al. [13], attaining up to a 95% sensitivity rate. Athinarayanan et al. [14] proposed a system with multiple stages proposed for cancer diagnosis and nuclei extraction. The Pap smear images were first treated to ensure noise removal during the preprocessing stage. These Pap smear images with little noise were used to extract texture features. The suggested system’s classification phase came next, using RBF and kernel-based SVM classification with these extracted features. The classification step achieves an accuracy above 94%. On the other hand, Plissiti et al. [15] applied spectral clustering and fuzzy C-means methods for the classification of cervical cells, focusing only on the features extracted from the nucleus, ignoring the cytoplasm. The best classification, 90.58%, was obtained with K-PCA (Gaussian kernel). Mbaga et al. [16] used an SVM classifier in two kernel functions: the radial basis and polynomial kernel functions. The performance of the classifier when using the polynomial kernel was much better and higher when compared to the Gaussian kernel; the best accuracy attained with the polynomial kernel was 97.02% at a polynomial degree value of d = 5. Previous classification techniques have depended on manually extracted features. Zhang et al. [17] introduced a method to classify cervical cells based on deep features using ConvNets CNN. Their method was evaluated on both Pap smear and LBC datasets. When applied to the Herlev benchmark Pap smear dataset and evaluated using five-fold cross-validation, results showed that classification accuracy was 98.3%. An automated, comprehensive machine-learning technique has been proposed by Malli et al. [18]. The proposed technique gives the color and shape features of the nucleus and cytoplasm of the cervix cell. KNN and neural networks were trained with these features, and then unknown cervix cell samples were classified. Results showed an accuracy of 88.04% for KNN and 54% for ANN.

In 2020, Wang et al. [19] designed a PsiNet-TAP network to classify Pap smear images. It was optimized by modifying the convolution layer and pruning some convolution kernels that could interfere with the target classification task. The network was tested on 389 cervical Pap smear images, and the method achieved an accuracy of more than 98%. Ghoneim et al. [20] proposed a system based on a CNN-extreme-learning-machine (ELM) and investigated autoencoder (AE)-based classifiers and multi-layer perceptron (MLP), alternatives to the ELM. Experiments performed using the Herlev database achieved 99.5% accuracy for the detection problem (two classes) and 91.2% for the classification problem (seven classes). Alquran et al. [21] proposed an ensemble machine-learning model with deep learning features extracted using ResNet 10 beside to features selection method. The best accuracy achieved for seven classes was 92%. In another study, Chen et al. [22] suggested using lightweight hybrid loss-constrained CNNs for the classification of cervical cells at the finest level. The proposed combined supervision of hybrid loss function improved the CNNs’ ability in categorizing cervical cells. ShufflenetV2 and GhostNet both achieved satisfactory classification using this approach (96.18% accuracy, 96.30% precision, 96.23% recall, and 99.08% specificity for ShufflenetV2 and 96.39% accuracy for GhostNet) [22]. Waly et al. proposed a system that uses intelligent deep CNN by using biomedical Pap smear images. First, they removed noise using a Gaussian filter. Then, they segmented the nuclei using the Tsallis entropy technique with dragonfly optimization and used SqueezeNet to extract features. Finally, classification was performed using weighted ELM. They achieved high accuracy, at 97.81% [23]. Fekri-Ershad and Ramakrishnan [24] suggested an effective multi-stage method for detecting cervical cancer in Pap smear images. First, the cytoplasm, including the nucleus, is removed from the intracellular fluid in the cervix cell using a straightforward thresholding method. The local textural elements are then described using a texture descriptor called modified uniform local ternary patterns (MULTP). Then, to classify the Pap smear images, a multi-layer feed-forward neural network is optimized. Recently, Deepa and Rao (2023) proposed a useful assistive tool for radiologists and clinicians to detect overlapped cervical cells using the CNN model with Rectified Linear Unit (ReLU) classifier. The model used 917 Pap smear cell images from the Herlev Dataset for training and testing, with 96% accuracy [25].

Reviews of the literature indicate that early cancer detection is essential for the treatment process in general; thus, early detection is also crucial for cervical cancer treatment. The Pap smear test is an important screening test for detecting cervical cancer, while the gold standard for diagnosing cervical cancer remains biopsy of cervical tissue. The Pap smear test has limitations. It may not detect all cases of cervical cancer or precancerous lesions. Therefore, regular screening is essential to maximize its effectiveness. The use of artificial intelligence techniques in medical imaging, such as ML, DL, and CNN, has gained popularity in recent years [26]. One constraint, though, is posed by morphological alterations and how they are intertwined with the cells’ structural components. Algorithms using DL and ML have significantly improved the healthcare sector. Moreover, deep learning advancements have produced neural network algorithms that can now compete with humans in computer vision-like image classification. Previous studies have focused on whole Pap smear images or on extracted nuclei to detect cervical cancer. This study focused instead on the area surrounding the nucleus, which is affected by the presence or absence of cancerous cells. At times, the nucleus is not clear in Pap smear images. Therefore, the surrounding regions can significantly contribute to the classification task. This study excluded the nucleus from Pap smear images and then classified them based on automated extracted features using deep-learning algorithms, feeding the features into the ML algorithm to discriminate among seven classes of Pap smear images.

## 3. Materials and Methods

**Figure 2:**
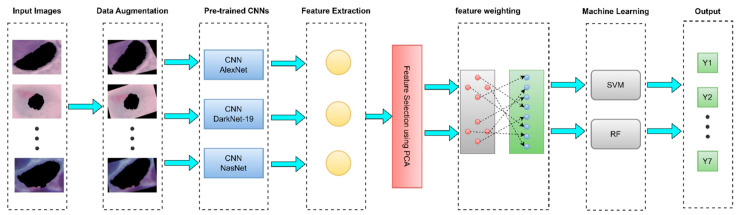
Block diagram of the proposed methodology.

The first step was preparing the image dataset for the system implementation. The approach used for Pap smear image classification is shown in Figure 2: first, the deep-learning algorithms, feature extraction, and feature reduction; then, training the SVM kernel and RF kernel; and finally, the diagnosis of the Pap smear images for all scenarios whole image, without a nucleus, and only nucleus.

### 3.1. Dataset

**Figure 3:**
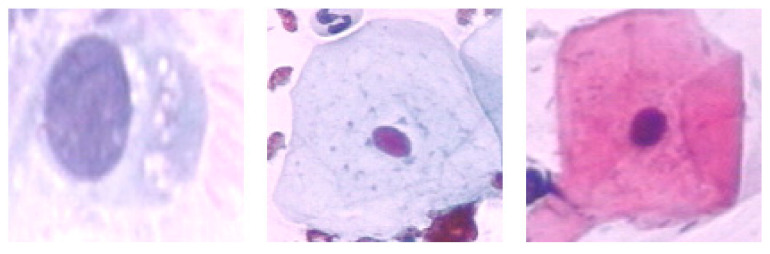
Normal classes for whole cell.

**Figure 4:**
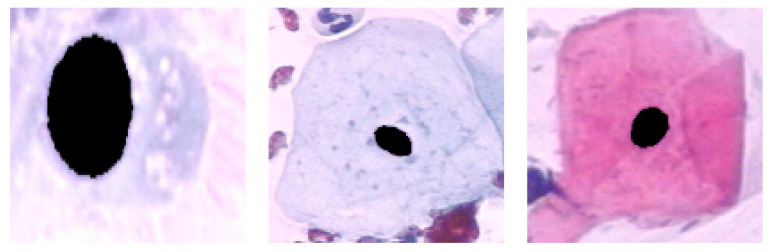
Normal classes with Nucleus exclusion.

**Figure 5:**
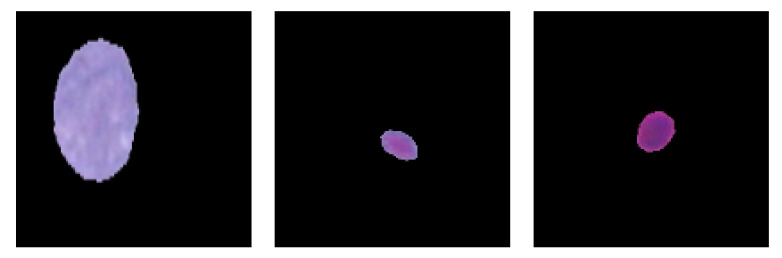
Normal classes with exclusion surrounding region.

**Figure 6:**
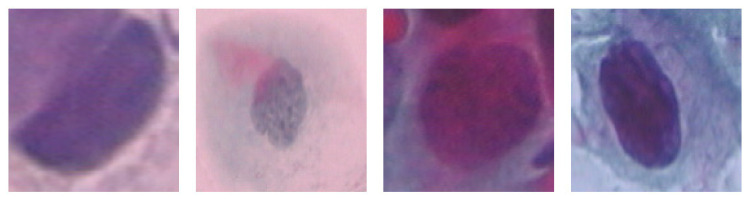
Abnormal classes for whole cell.

**Figure 7:**
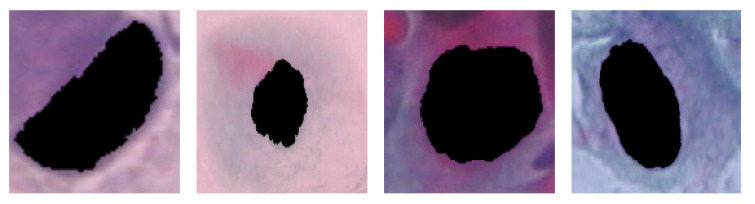
Abnormal Classes with Nucleus exclusion.

**Figure 8:**
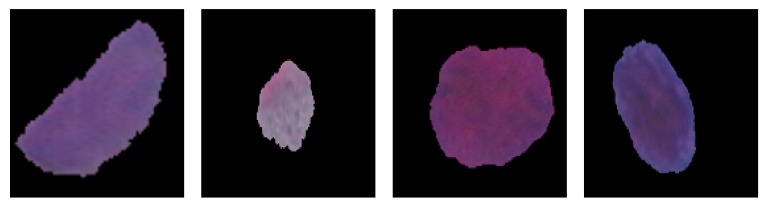
Abnormal Classes with exclusion surrounding region.

This study used the Herlev Pap smear dataset, consisting of 917 cell images, with each image containing one nucleus. To determine the effect of the surrounding region of the nucleus, the nucleus was excluded from each cell image. The dataset was collected by Herlev University Hospital (Denmark) and the Technical University of Denmark [27]. Table 1 shows the distribution of cervical images for each class; three are normal, and the rest are types of abnormal classes. Figure 3, Figure 4 and Figure 5 show the normal classes for whole cell, with nucleus exclusion, and with exclusion surrounding region, respectively, while Figure 6, Figure 7 and Figure 8 represent the abnormal classes for whole cell, with nucleus exclusion, and with exclusion surrounding region, respectively.

### 3.2. New Image Augmentation

A technique called data augmentation involves adding copies of already existing data that have been significantly altered or product synthetic data that have been generated from already existing data [28]. Data augmentation causes regularization and reduces overfitting. This study applied image augmentation to the dataset by rotating images at random angles between [−20, 20], scaling images using various scale factors within [0.1, 1], and translating images in both X and Y directions between [−1, 1]. The count of images before and after augmentation is shown in Table 2.

The augmentation procedure is performed in the same manner for all scenarios to ensure that the comparison is reasonable and under the same conditions. The number of images increases to 1400 (200 for each class for all cases).

### 3.3. Deep Learning Features

Deep learning techniques are widely employed across a variety of fields to address a wide range of complicated issues, including computer vision, speech recognition, and image processing. The most frequently used DL algorithms are CNN and Recurrent Neural Networks (RNN) [29]. The necessity for manual feature extraction is eliminated by the ability of CNN to extract the features of the input image. To extract the most representative features, three pre-trained CNN models are applied. The DL structures used were trained on the ImageNet dataset to differentiate among 1000 natural classes. Transfer learning methods were employed to render these structures compatible with the intended problem, which focused on identifying seven categories of cervical cells [26]. Transfer learning appeared by changing the image input size to be suitable with the input layer of each one and eliminating the last completely connected layer to leave seven neurons for seven classes. AlexNet, DarkNet-19, and Nasnet were used for feature extraction.

#### 3.3.1. AlexNet

**Figure 9:**
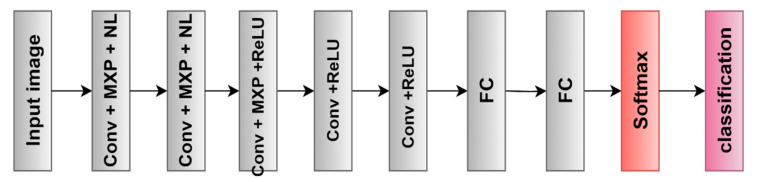
AlexNet architecture [30].

DL algorithms assist in automatically extracting features from a large dataset. The first CNN to use a GPU to improve performance was AlexNet. Five convolutional layers, three max-pooling layers, two normalization layers, two fully connected layers, and a single softmax layer make up the AlexNet architecture. The input size is typically stated as 224 × 224 × 3, but, due to padding, it comes out to be 227 × 227 × 3. To be appropriate through the first input layer, all images were modified [30]. Here, the top seven features are automatically extracted using the transfer learning strategy (Figure 9).

#### 3.3.2. DarkNet-19

**Figure 10:**
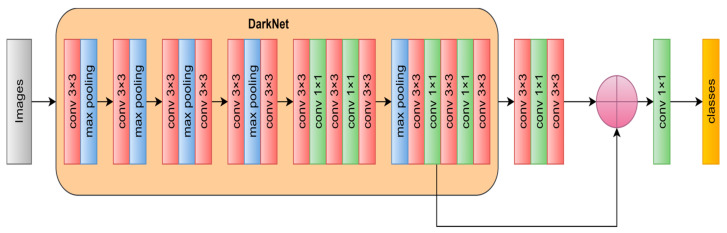
The structure of DarkNet-19 [32].

The CNN with 19 layers is known as DarkNet19. DarkNet19 consists of five max-pooling layers and 19 convolutional layers, ranging from many 1 × 1 CL to the smallest triangle parameters comprising 3 × 3. The deep feature was extracted at this stage using DarkNet19. Additionally, the input layer image size for DarkNet19 is 256 × 256. Figure 10 displays the structure of the neural network of DarkNet19 [31].

#### 3.3.3. NasNet

**Figure 11:**
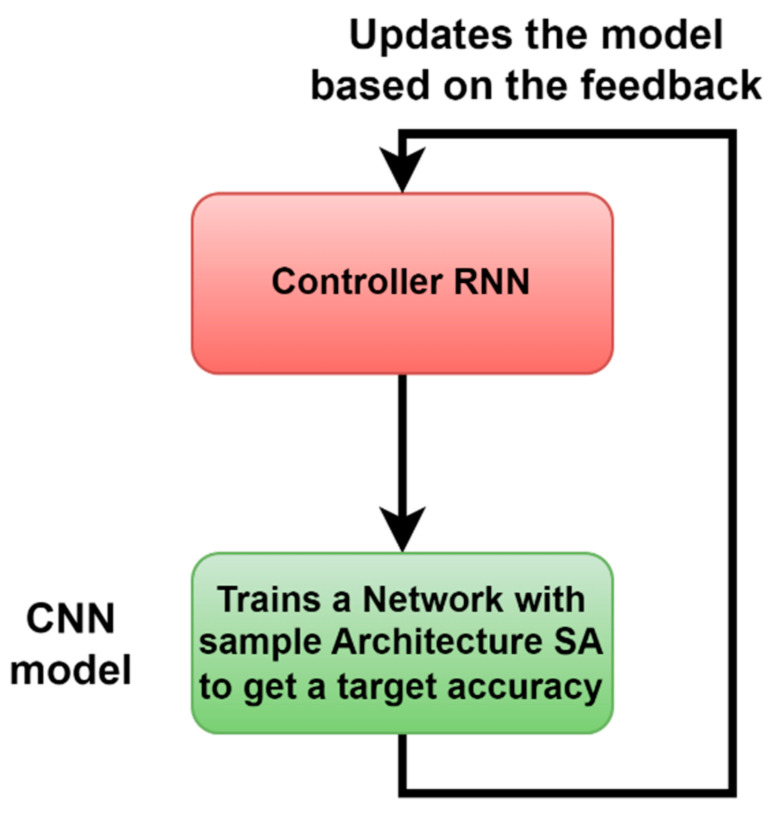
Controller RNN in NASNet architecture [33].

NasNet is a new, optimizable deep neural network based on the reinforcement learning approach, which is the parent artificial intelligence (AI), making corrections and modifications of the weights, number of layers regularization methods of the child of AI. Its architecture is composed of two main blocks: controller recurrent neural network (CRNN) and CNN. There are three different versions: A, B, and C. Figure 11 demonstrates its basic idea [33]. In this stage, transfer learning is used, and the last layers are used to extract seven features.

### 3.4. Principal Component Analysis (PCA)

The fundamental principle behind utilizing PCA as a feature selection method is to choose variables based on the magnitude (from biggest to lowest in absolute values) of the coefficients. Each variable contributes differently to each of the primary components, which are ranked in importance by their explained variance [34].

All previously pre-trained CNNs’ 21 collected features are processed, and the 10 most promising features are chosen for additional processing and diagnosis. These independent features came from six NasNet features, two DarkNet-19 features, and the remaining features from AlexNet. The features reduction method is described in Table 3, and works by examining the number of features from each pretrained model, the total extracted features from all used CNN models by combination the extracted features, applying PCA, and then the most significant features by displaying the number of candidates features from each model.

### 3.5. Feature Weighting Method

The feature weighting aims to determine the relative value of each feature in relation to the classification job and provide it an appropriate weight. A vital feature would be given a higher weight than a less significant or irrelevant feature if the weighting were conducted appropriately. Feature weighting employs a continuous value and, as a result, has a higher degree of detail in assessing the relevance of feature rather than making a binary decision. It works better for tasks where certain features are more important than others [35].

Using the strategy of ant and lion hunting, Seyedali Mirjalili introduced ALO in 2015 [36]. The ALO algorithm is used to simultaneously obtain the best feature weights and parameter values for neural networks. The five main steps of this hunting strategy are the random walk of agents, trapping ants in a trap, building traps, tearing them down, and catching prey. Numerous ant lions and ants with randomly placed locations are present throughout the search area. Ants will travel randomly throughout the search area, potentially going through or around the traps, to mimic how ant lions and ants interact. The more skilled individual or “ant lion” will dig a deeper, sharper pit, and capture more ants. Ant lions, specifically the elite (best) ant lion, influence the random movements of ants. To ensure the variety and optimization ability of the algorithm, ants will change their positions according to ant lion and elite ant lion [37,38].

The Particle Swarm Optimization (PSO) starts by randomly initializing the particle positions. The fitness value of each particle was then determined based on the assessment function. Until the desired state is attained, this procedure will keep iterating. Each particle is impacted by two values during each iteration. The first is best value any particle has ever attained. The second value is the best overall value that all particles in the sample have. The particles updated their velocity and positions after achieving the two best results [39].

### 3.6. Classification

#### 3.6.1. Support Vector Machine

**Figure 12:**
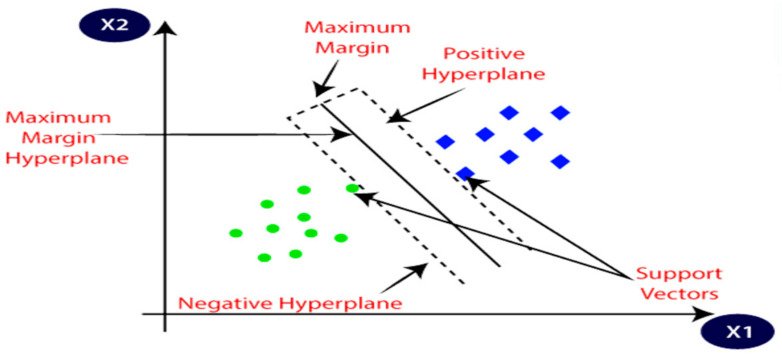
The principal operation of SVM classifier [40].

SVM is a well-known ML approach that is used for both classification and regression. The SVM algorithm’s objective is to establish the best line or decision boundary that can divide n-dimensional space into classes, allowing the quick classification of new data points. This optimal decision boundary is called a hyperplane. SVM selects the extreme vectors and points that aid in the creation of a hyperplane. Support vectors, which are used to represent these extreme instances, form the basis of the SVM method. Consider the diagram below, where a decision boundary or hyperplane is used to categorize two distinct categories. SVM uses the kernel trick, which maps the features to higher dimensional space to find the appropriate training model that can be generalized. The polynomial kernel is exploited in this study [40,41,42]. Figure 12 illustrates the principle of SVM.

#### 3.6.2. Random Forest (RF)

**Figure 13:**
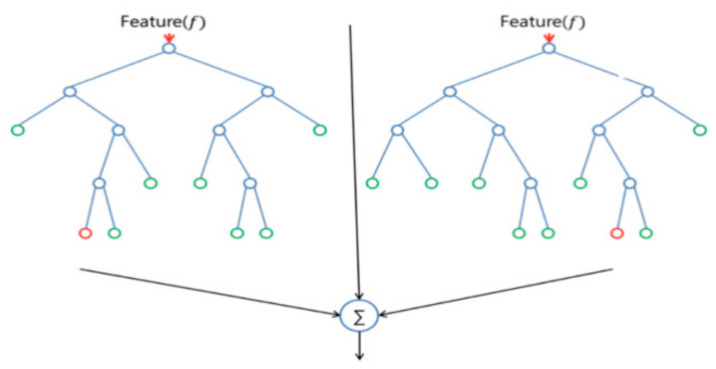
The principal operation of RF classifier [43].

The decision tree (DT) is the random forest classifier’s unit block. Each RF is made up of numerous independent DTs that work together to form an ensemble classifier. Several decision trees are grown and combined in the RF model to form a “forest.” Another kind of algorithm used to categorize data is the decision tree. In the simplest terms, it works like a flowchart that shows a clear path to a choice or result; it begins at one point and branches out in two or more ways, with each direction giving a range of potential possibilities. While the trees are developing, the random forest adds more randomness to the model. When dividing a node, it looks for the best feature from a random subset of features rather than the most crucial one. A better model is often produced because of the great diversity caused by this process. Figure 13 describes the principal operation of RF classifier [43].

## 4. Results

### 4.1. Ant Lion Optimization

**Figure 14:**
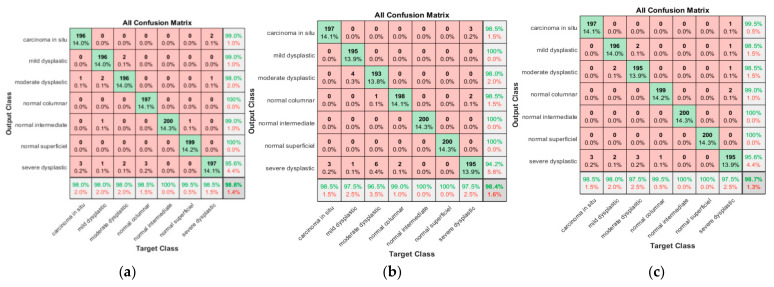
Confusion matrix for ALO-RF for various scenarios; (**a**) For nucleus only, (**b**) Whole cell exclusion, (**c**) for nucleus only.

Using ALO weighting algorithm after feature selection, we conducted three experiments, where once the image was whole, another time we segmented the nucleus, and the last one we excluded the nucleus. This method’s main steps include data augmentation, automatic feature extraction utilizing transfer learning from several CNN structures, feature reduction via PCA, and a novel feature weighting technique. The 10 most significant features were extracted. These graphical features were ordered as follows: six features from NasNet, two from Darknet-19, and two from AlexNet. This distribution of the most significant features demonstrated that the NasNet extracted the most relevant features for classes and that is compatible with its deep structure. The K-fold cross validation techniques are used. Both SVM and RF were used to classify the images into seven classes based on the selected automated graphical features. A confusion matrix is a table that lists how many predictions a classifier made correctly and incorrectly. It is employed to evaluate a classification model’s effectiveness. With the measurement of performance metrics like accuracy, precision, recall, and F1-score, it can be utilized to assess a classification model’s effectiveness [38]. Figure 14 illustrates the results of the confusion matrix of RF with ALO weighting method.

By using the corresponding matrices [42], the evaluation of the classifiers are performed.

$$Accuracy=\frac{TP+TN}{TP+FN+FP+TN}$$

$$Precision=\frac{TP}{TP+FP}$$

$$Sensitivity=\frac{TP}{TP+FN}$$

$$F1-Score=\frac{2\times Precision\times Sensitivity}{Precision+Sensitivity}\;$$

Figure 14a shows that 196 carcinomas in situ cases were classified correctly, with three cases misclassified as severe and one as dysplastic. The sensitivity obtained was 98%, and the positive predictive value was 99%. From 200 mild dysplastic cases, 196 were classified correctly, with a true positive rate of 98%. There were four misclassified cases: two as moderate dysplastic and two as normal and severe dysplastic, respectively. The precision of the mild category was 99%. The results for moderate dysplastic were 98% as recall and PPV was 98%. For normal columnar, the sensitivity was 98.5%, with three cases misclassified cases one as severe dysplastic. On the other hand, the precision was 98%. However, promising results were achieved in normal intermediate normal columnar and normal superficial classes, with almost 100% recall and precision. The last class was severe superficial; here, the lowest sensitivity reached 95.6%, with three cases misclassified as carcinoma in situ, one misclassified as mild dysplastic, two misclassified as moderate dysplastic, and three as normal intermediate. The proposed approach provides promising results, with an accuracy of 98.6% and an overall misclassification rate of 1.4%.

Figure 14b shows that one hundred and ninety-seven carcinomas in situ cases were classified correctly, with three cases misclassified as severe dysplastic. The sensitivity and precision obtained were 98.6%. Four mild dysplastic cases were misclassified as moderate dysplastic and 195 out of 200 mild dysplastic cases were classified correctly, with a true positive rate of 97.5. The precision of the mild category was 100%. The results for moderate dysplastic were 96.5% as recall, with six misclassified cases as severe and one as columnar. In addition, the PPV was 98%, where four mild dysplastic cases were incorrectly classified as moderate. For normal columnar, the sensitivity was 99%, with two cases misclassified as severe. On the other hand, the precision was 98.5%, with two cases classified as severe and one as moderate dysplastic. However, promising results were achieved in both normal intermediate and normal superficial classes, with 100% recall and precision. The last class was severe superficial; here, the sensitivity reached to 97.5%, with three cases misclassified as carcinoma in situ and two misclassified as normal columnar. However, the moderate precision obtained in the severe dysplastic class reached 94.2%. The proposed approach provides promising results, with an accuracy of 98.4% and an overall misclassification rate of 1.6%

Figure 14c shows that 190 of 200 carcinomas in situ cases were classified correctly, with three cases misclassified as sever dysplastic. The sensitivity obtained was 98.5%, and the positive predictive value was 99.5%. Of 200 mild dysplastic cases, 196 were classified correctly, with a true positive rate of 98%, with four misclassified cases: two as moderate dysplastic and the other two as severe dysplastic. The precision of the mild category was 98%. The results for moderate dysplastic were 97.5% as recall and PPV was 98.5%, where two mild dysplastic cases were incorrectly classified as moderate and another one as severe. For normal columnar, the sensitivity was 99.5%, with one case misclassified as severe. On the other hand, the precision was 99%, with two cases classified as severe. However, promising results were achieved in both normal intermediate and normal superficial classes, with 100% recall and precision. The last class was severe superficial, where sensitivity reached 97.5%, with five cases misclassified as carcinoma in situ, mild, moderate, and two misclassified as normal columnar. The proposed approach provides promising results, with an accuracy of 98.7% and an overall misclassification rate of 1.3%.

**Figure 15:**
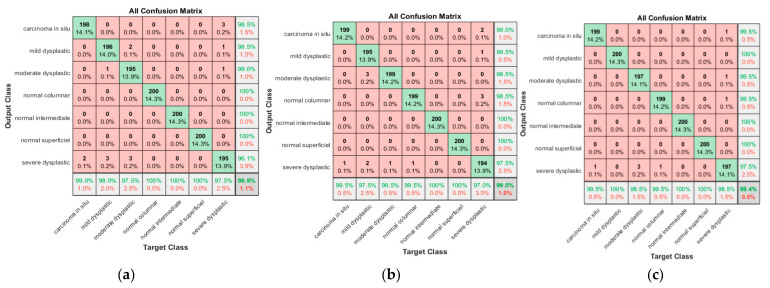
Confusion matrix for ALO-SVM for various scenarios; (**a**) for nucleus exclusion (**b**) For Whole cell, (**c**) for nucleus only.

As is clear from Figure 15a, 198 cases were classified correctly among 200 cases, with two cases misclassified as severe dysplastic. The obtained sensitivity was 99%, and the positive predictive value was 98.5%. Two severe dysplastic cases were misclassified as carcinoma in situ. Of 200 mild dysplastic cases, 196 were classified correctly, with a true positive rate of 98%; there were only four misclassified cases (one as moderate and three as severe). The precision of the mild category was 99.5%. The maintained results for moderate dysplastic are 97.5% as recall and two misclassified case, as mild and three cases as severe dysplastic. In addition, the PPV was 99%; one severe dysplastic case and one mild case were classified as moderate. For normal columnar, normal intermediate, and normal superficial, the promising results were obtained with the highest sensitivity and a precision of 100%. The last class was severe superficial, with a sensitivity reaching 97.5%, with three cases being misclassified as carcinoma in situ, one as normal columnar, and one as moderate. However, the precision in the severe dysplastic class reached 96.1%. The proposed approach provided promising results, with an accuracy of 98.9% and an overall misclassification rate reaching 1.1%.

As is clear from Figure 15b, 199 carcinomas in situ cases were classified correctly among 200 cases, with one case misclassified as severe dysplastic. The obtained sensitivity was 99.5%, and the positive predictive value was 99%. While two severe dysplastic was misclassified as carcinoma in situ, 195 mild dysplastic cases were classified correctly, with a true positive rate of 97.5%; there were only five misclassified cases: three as moderate dysplastic and the other as severe dysplastic. The precision of the mild category was 99.5%. Just one severe case was misclassified as mild dysplastic. The maintained results for moderate dysplastic are 99.5% as recall and one misclassified case as mild. In addition, the PPV was 98.3%, where three mild was incorrectly classified as moderate. For normal columnar, the sensitivity was 99.5%, with one case misclassified as severe. Nevertheless, the precision obtained in the columnar class was 98.5%, with three severe cases being misclassified as normal columnar. However, promising results were obtained in both normal intermediate and normal superficial classes, with 100% recall and precision. The last class was severe superficial, with a sensitivity reaching 97%, with one case being misclassified as carcinoma in situ, two as mild, with three misclassified cases as normal columnar. However, the precision obtained in the severe dysplastic class reached 97.5%. The proposed approach provided promising results, with an accuracy of 99% and an overall misclassification rate reaching 1%.

As is clear from Figure 15c, 199 carcinoma in situ cases were classified correctly from 200 cases, with one case misclassified as severe dysplastic. The obtained sensitivity was 99.5%, and the positive predictive value was 99%. One severe dysplastic was misclassified as carcinoma in situ. The promising results in mild dysplastic cases showed 200 cases classified correctly, with a true positive rate of 100%. The precision of the mild category was 100%. The maintained results for moderate dysplastic are 98.5% as recall, with three cases misclassified as severe. In addition, the PPV was 99.5%, where one severe dysplastic case was incorrectly classified as moderate. For normal columnar, the sensitivity was 99.5%, with one case misclassified as severe and one as carcinoma in situ. The precision obtained in the columnar class was 99.5%. Promising results were obtained in both normal, intermediate, and normal superficial classes, with 100% recall and precision. The last class was severe superficial, with a sensitivity reaching 98.5%. Three cases were misclassified as carcinoma in situ, one as moderate, and one as normal columnar. The lowest precision obtained in the severe dysplastic class reached 97.5%, where five cases were misclassified as severe dysplastic: three from moderate, one from carcinoma in situ, and one from normal columnar. The proposed approach provided promising results, with an accuracy of 99.4% and an overall misclassification rate reaching 0.6%.

### 4.2. Particle Swarm Optimization

The same selected features are weighted swarm optimization method for various scenarios. The following confusion matrices illustrated the outcomes of the RF and SVM.

**Figure 16:**
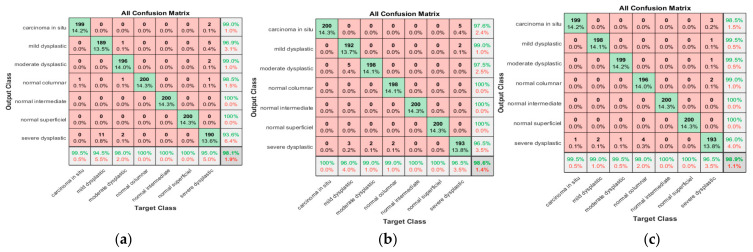
Confusion matrix for SVM for various scenarios; (**a**) for nucleus only (**b**) For Whole cell, (**c**) for nucleus exclusion.

Figure 16a shows that 199 carcinomas in situ cases were classified correctly, with one case misclassified as sever dysplastic. The sensitivity obtained was 99.5%, and the positive predictive value was 99%. Of 200 mild dysplastic cases, 189 were classified correctly, with a true positive rate of 94.5%; there were eleven cases misclassified as severe dysplastic. The precision of the mild category was 96.9%. The best achieved results in sensitivity term for moderate dysplastic, normal columnar, and normal superficial were 100%; the same value in precision was achieved, but the moderate achieved 98.5%, where three case were misclassified as moderate (one from carcinoma in situ, one from normal columnar, and the last one comes from severe dysplastic). The last class was severe dysplastic; here, the sensitivity reached 95.0%, with five cases misclassified as mild, two as carcinoma in situ, two misclassified as moderate dysplastic, and one as normal intermediate. The proposed approach provides promising results, with an accuracy of 98.1% and an overall misclassification rate of 1.9%.

As is clear from Figure 16b, 200 carcinomas in situ cases were classified correctly among 200 cases, with an obtained sensitivity of 100%; the positive predictive value was 97.6%. Five severe dysplastic was misclassified as carcinoma in situ, one hundred ninety-two mild dysplastic cases were classified correctly, with a true positive rate of 96%; three cases were misclassified as severe dysplastic. The precision of the mild category was 99.0%; two severe class were misclassified as mild dysplastic. The maintained results for moderate dysplastic are 99% as recall and two misclassified cases as severe. In addition, the PPV was 97.5%, with five mild cases incorrectly classified as moderate. For normal columnar, the sensitivity was 99.0%, with two cases misclassified as severe. Nevertheless, the precision obtained in the columnar class was 100%. Promising results were obtained in both normal intermediate and normal superficial classes, with 100% recall and precision. The last class was severe superficial, with a sensitivity reaching 96.5%, with five cases being misclassified as carcinoma in situ and two as mild. The precision obtained in the severe dysplastic class reached 96.5%. The proposed approach provided promising results, with an accuracy of 98.6% and an overall misclassification rate reaching 1.4%.

Figure 16c shows that 199 of 200 carcinomas in situ cases were classified correctly, with one case misclassified as severe dysplastic. The sensitivity obtained was 99.5%, and the positive predictive value was 98.5%. Of 200 mild dysplastic cases, 198 were classified correctly, with a true positive rate of 99%; two misclassified cases were classified as severe dysplastic. The precision of the mild category was 99.5%. The results for moderate dysplastic were 99.5% as recall and PPV was 99.5%, where one mild dysplastic case was incorrectly classified severe. For normal columnar, the sensitivity was 98%, with four cases misclassified as severe. The precision was 99%, with two cases classified as severe. Promising results were achieved in both normal intermediate and normal superficial classes, with 100% recall and precision. The last class was severe superficial. Sensitivity reached 96.5%, with three cases misclassified as carcinoma in situ, one as mild, one as moderate, and two misclassified as normal columnar. The proposed approach provides promising results, with an accuracy of 98.9% and an overall misclassification rate of 1.

**Figure 17:**
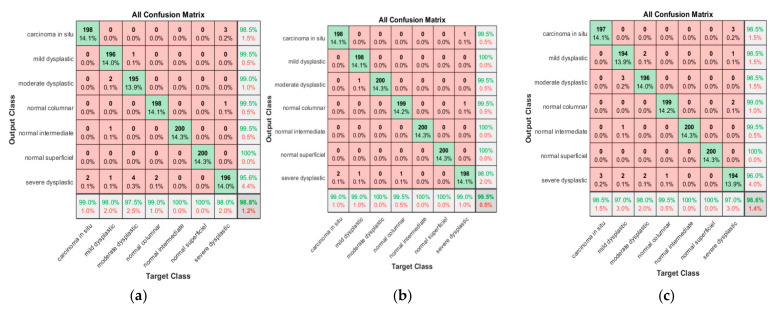
Confusion matrix for SVM for various scenarios; (**a**) for nucleus exclusion, (**b**) For Whole cell, (**c**) for nucleus only.

As is clear from Figure 17a, 198 cases were classified correctly among 200 cases, with two cases misclassified as severe dysplastic. The obtained sensitivity was 99%, and the positive predictive value was 98.5%. Three severe dysplastic were misclassified as carcinoma in situ. Of 200 mild dysplastic cases, 196 were classified correctly, with a true positive rate of 98%. There were four misclassified cases (two as moderate, one as normal intermediate, and one as severe). The precision of the mild category was 99.5%. The maintained results for moderate dysplastic are 97.5% as recall and four cases were misclassified as severe dysplastic. The PPV was 99%, and two mild cases were classified as moderate. For normal columnar the sensitivity is 100% and precision is 99.5%. For normal intermediate and normal superficial, promising results were obtained with the highest sensitivity and precision of almost 100%. The last class was severe superficial, with a sensitivity reaching 98%, with three cases being misclassified as carcinoma in situ and one as normal columnar and one as moderate. The precision in the severe dysplastic class reached 96.6%. The proposed approach provided promising results, with an accuracy of 98.8% and an overall misclassification rate reaching 1.2%.

As is clear from Figure 17b, 198 carcinomas in situ cases were classified correctly among 200 cases, with obtained sensitivity was 99%; the positive predictive value was 99.5%. While one severe dysplastic was misclassified as carcinoma in situ, 198 mild dysplastic cases were classified correctly, with a true positive rate of 99%, with only one misclassified as severe dysplastic. The precision of the mild category was 100%. The maintained result for moderate dysplastic are 100% as recall. The PPV was 99.5%, where one mild case was incorrectly classified as moderate. For normal columnar, the sensitivity was 99.5%, with one case misclassified as severe. The precision obtained in the columnar class was 99.5%. Promising results were obtained in both normal intermediate and normal superficial classes, with 100% recall and precision. The last class was severe superficial, with a sensitivity reaching 99%, with one case being misclassified as carcinoma in situ and one case as normal columnar. The precision obtained in the severe dysplastic class reached 98%. The proposed approach provided promising results, with an accuracy of 99.5% and an overall misclassification rate reaching 0.5%.

Figure 17c shows that 197 of 200 carcinomas in situ cases were classified correctly, with three cases misclassified as severe dysplastic. The sensitivity obtained was 98.5%, and the positive predictive value was 98.5%. Of 200 mild dysplastic cases, 194 were classified correctly, with a true positive rate of 97%; two cases were misclassified as severe dysplastic and three misclassified cases as moderate. The precision of the mild category was 98.5%. The results for moderate dysplastic were 98.0% as recall and PPV was 98.5%, with three mild dysplastic case incorrectly classified as moderate. For normal columnar, the sensitivity was 99.5%, with one case misclassified as severe. The precision was 99%, with two cases misclassified as severe. Promising results were achieved in both normal intermediate and normal superficial classes, with almost 100% recall and precision. The last class was severe superficial. Sensitivity reached 97%, with three cases misclassified as carcinoma in situ, one as mild, and two misclassified as normal columnar. The proposed approach provides promising results, with an accuracy of 98.6% and an overall misclassification rate of 1.4%.

It is obvious from the previous discussion that the performance of Swarm optimization after feature selection is better than ALO, either in SVM or RF classifiers. The best scenario is obtained in nucleus exclusion case. This indicates the effectiveness of the surrounding region on the diagnosis of the cervical cells.

As is clear from previous results, the combination of automated features with the ML classifiers beside various weighting algorithms was capable of distinguishing among various classes of cervical cells, thus establishing the effectiveness of focusing the difference of using whole cell, nucleus exclusion, and nucleus only. The results of using the two types of classifiers with deep features with NasNet and the most common pre-trained CNNs AlexNet and DarkNet-19 beside two types of optimization techniques are compared with the literature. Table 4 represents the study, the proposed method, number of classes, the data set used, and their results.

As is clear from the table, the proposed method is capable of distinguishing among the seven classes. This study encourages the use of cytoplasm features instead of whole cell or nucleus region, which is easier than studying the nuclei in Pap smear cells. This study may lead to a good approach in cases where the shape of the nucleus cannot be handled or is unreachable by various image processing techniques and in which taking patches from the cytoplasm and passing the proposed model can expect or guide the class of the Pap smear image sample. The proposed approach achieved the highest accuracy among literature either using whole cell or without nucleus. The maintained results approved the ability of the proposed method to diagnose cervical cells into seven classes without further processing to segment the nucleus. The diagnosis can be performed using cytoplasm or whole cell. This approach yields to preserves time and effort.

## 5. Conclusions

A new method that may lower the mortality rate from cervical cancer among women is the detection of the disease using Pap smear images. Determination of the type of cervical cancer depends on the cell nuclei visible in a Pap smear image. Sometimes, however, the nuclei may be difficult to visualize, in which case the area surrounding the nucleus may help in the diagnosis of Pap smear images. This study aims to compare the performance of using whole cell, nucleus region, or cytoplasm region only in order to distinguish the type of cervical cell, due to some limitations on segmentation of nucleus and the challenges of delineating the nucleus region. Therefore, the proposed approach focuses on comparing the effectiveness of three scenarios: whole cell, surrounding region, and nucleus only. The proposed approach does not require pre-processing of the images. This study directed the most significant automated features at the region surrounding the nucleus, nucleus, and whole cell. Based on our knowledge, this is the first study comparing the various types of regions for seven classes beside utilizing various optimization algorithms (AntLion and swarm optimization methods). The obtained results enhance the capability of the researcher to take a patchy image from the cytoplasm and diagnose the pap smear images without requiring additional processing around the nucleus features or whole cell properties. The proposed system achieved an accuracy for seven classes of 99.5% using the SVM classifier and swarm optimization, after selecting the ten most significant deep features, the weighting algorithm optimize the effectiveness of each feature to distinguishing of various classes. These features are intrinsic to each class because they are automated, deeply extracted, graphical features. The utilization of feature-reduction techniques and weighting algorithms enhance the accuracy beside to complexity and computation time. This automated system may help physicians when the nucleus is not clear to include the whole cell or the surrounding regions only. This CAD system can be beneficial to the doctors and physicians in rural countries.

## Figures and Tables

**Table 1:** Distribution of data.

Cell Type (Seven Classes)	Counts
Superficial squamous epithelial (Normal)	74
Intermediate squamous epithelial (Normal)	70
Columnar epithelial (Normal)	98
Mild squamous non-keratinizing dysplasia (Abnormal)	182
Moderate squamous non-keratinizing dysplasia (Abnormal)	146
Severe squamous non-keratinizing dysplasia (Abnormal)	197
Squamous cell carcinoma in situ intermediate (Abnormal)	150

**Table 2:** Distribution of the dataset after augmentation.

Cell Type (Seven Classes)	Before Augmentation	After Augmentation
Superficial squamous epithelial (Normal)	74	200
Intermediate squamous epithelial (Normal)	70	200
Columnar epithelial (Normal)	98	200
Mild squamous non-keratinizing dysplasia (Abnormal)	182	200
Moderate squamous non-keratinizing dysplasia (Abnormal)	146	200
Severe squamous non-keratinizing dysplasia (Abnormal)	197	200
Squamous cell carcinoma in situ intermediate (Abnormal)	150	200

**Table 3:** Number of features before and after PCA.

Pre-Trained Model	Number of Extracted Features	The Combination of Whole Features	Using PCA
AlexNet	7	21 features	2
DarkNet-19	7		2
NasNet	7		6
	21 features	21 features	10 features

**Table 4:** The comparison between the proposed study and the literature.

Ref.	Method	Number of Classes	Type of Dataset	Result
[11]	Shape and gray co-occurrence features; they employed the adaptive neuro fuzzy inference system	Normal and Abnormal	Herlev	Accuracy reached 92.68%
[12]	Utilized texture features for processed Pap smear images	Normal and Abnormal	Herlev	Accuracy reached 99.1%
[14]	Texture features of nucleus	Normal and Abnormal	Herlev	Accuracy reached 94%
[15]	Spectral features of the nucleus with K-PCA	Normal and Abnormal	Herlev	Accuracy reached 90.58%
[17]	Deep features, using ConvNets CNN	Normal and Abnormal	Herlev	Accuracy reached 98.3%
[18]	Color and shape features of nucleus and cytoplasm of the cervix cell beside machine learning	Normal and Abnormal	Herlev	Accuracy reached 88.04%
[20]	CNN-ELM-based system and an autoencoder (AE)-based classifiers and multi-layer perceptron (MLP),	Seven classes	Herlev	Accuracy reached 91.2%
[21]	Deep features with feature selection combined with ensemble machine learning model	Seven classes	Herlev	Accuracy reached 92%
[22]	Hybrid Loss-Constrained Lightweight Convolutional Neural Networks for Cervical Cell Classification	Seven classes	Herlev	Accuracy reached 96.39%
[23]	Optimal deep convolution neural network for cervical cancer diagnosis model	Seven classes	Herlev	Accuracy reached 97.81%
[24]	Uniform local ternary patterns and feed forward multilayer network optimized by genetic algorithm	Seven classes	Herlev	-
[25]	Classification of normal and abnormal overlapped squamous cells in Pap smear image	Seven classes	Herlev	Accuracy reached 96%
**This Study**	**Deep features with feature weighting method (ALO, PSO)**	Seven classes	Herlev	Accuracy reached **99.5%**

## Data Availability

The dataset analyzed during the current study was derived from the Herlev Pap Smear dataset, which consists of 917 manually isolated Pap smear cell images. This dataset has been publicly available online since 2006. It is available on the corresponding website: (http://mde-lab.aegean.gr/index.php/downloads) (accessed on 10 March 2021).
